# Awareness of Peri-Implantitis Among General Dental Practitioners in Southern Saudi Arabia: A Cross-Sectional Study

**DOI:** 10.7759/cureus.77923

**Published:** 2025-01-24

**Authors:** Sultan Alanazi

**Affiliations:** 1 Department of Preventive Dental Sciences, College of Dentistry, Najran University, Najran, SAU

**Keywords:** dental implant, gdps, peri-implantitis, peri-implant mucositis, probing

## Abstract

Aim and background: The success of dental implants is contingent upon various biological conditions, and any lapse in meeting these criteria can lead to complications such as peri-implantitis or implant failure. The objective of this research is to evaluate the level of understanding regarding peri-implant among general dentists practicing in Saudi Arabia.

Materials and methods: A quantitative approach was employed, utilizing an online questionnaire distributed to general dental practitioners (GDPs) in Saudi Arabia. The level of significance was predetermined at a p-value less than 0.05, and chi-square tests were performed utilizing IBM SPSS Statistics for Windows, Version 20 (Released 2011; IBM Corp., Armonk, New York, United States). For collected data, descriptive analysis was also applied.

Results: In general, just a few numbers of participants demonstrated adequate knowledge concerning peri-implantitis. Approximately 13.5% of respondents had undergone training in dental implants and engaged in probing around implants. Smoking and uncontrolled diabetes were identified as significant contributors to peri-implantitis by 18% and 12% of participants, respectively.

Conclusion: Noteworthy concerns arose as 18.7% of practitioners did not conduct probing around implants. No statistically significant differences were observed based on gender, experience, or training level among the participants. This underscores the importance of addressing these lapses through targeted education and training programs to optimize overall patient care.

## Introduction

Inflammation in the surroundings of soft tissues of an osseointegrated implant, in addition to supporting bone loss, is defined as peri-implantitis [1]. The occurrence rate of up to 47% for peri-implantitis has been reported [2]. Signs of peri-implantitis in one out of five implants appeared in a recent retrospective study over time [3]. Advancements in implant dentistry, now integrated into mainstream dental practice, have empowered dentists to enhance their patients' quality of life over the past decade [4]. Dental implants have emerged as the preferred treatment for both partially and completely edentulous patients. The proper implementation of this treatment modality by dentists has significantly enhanced the life quality of these patients. This has led to notable improvements in patients' appearance, health, and functionality, even amidst challenging circumstances [5].

There is a relatively high success rate with dental implant treatment, but complications are still present due to improper planning, wrong prosthetic and surgical execution, material failure, and improper maintenance [6]. Inadequate control of plaque, periodontitis, systemic diseases, and defects of soft tissue represent the primary risk factors associated with peri-implantitis [7]. Inflammation and bacterial infection in the tissues surrounding the implant can cause implant failure. About 14.6% of patients with implants show bone loss and inflammation in implant surroundings [8]. In the sixth workshop of the European Periodontology Association, which was conducted in 2008, it was noted that there was a prevalence range of 28-56% for peri-implantitis. Peri-implantitis and mucositis can affect the prognosis of dental implants [9]. Patients with these conditions can be classified into: (1) normal periodontium, (2) gingivitis related to peri-implant, and (3) marked periodontitis considered peri-implantitis. Risk assessment is also important and necessary for these patients. There is a basic necessity for examining the periodontium using adequate examination procedures in these patients [10]. Measuring of bleeding, depth of probing, and study of bone loss shown on radiographic analyses are required for the diagnosis of peri-implantitis [11]. The accurate diagnosis of peri-implantitis is crucial for determining the appropriate clinical management strategies [12]. Research conducted in specialized environments recognizes the involvement of general dental practitioners (GDPs) in diagnosing, referring, and managing patients with these conditions [13]. The regular monitoring of peri-implantitis by GDPs, as well as the organization of follow-up regimens tailored to patient feasibility, relies on the broad understanding of the symptoms and signs associated with peri-implant mucositis and peri-implantitis. Successful treatment results from early diagnosis and awareness of risk factors [14]. These adverse effects on the patient are prevented by the skillful efforts of the dental team, including the laboratory technicians, as well as by the acceptance of minor deviations from ideal esthetics, function, and shape by the patients [15]. A well-organized recall system is required for the practical maintenance of implant patients [16]. Maintenance of dental implants has two components, namely home and office care. The most common techniques, including in-home care maintenance, are flossing, tooth brushing, interdental brushes, and irrigation systems. Common techniques for office care maintenance are visual examination, scalers and curettes, debridement of hard and soft tissue deposits, and polishing. Plastic or carbon-tipped scalers are best for use to prevent any damage to the surface of the implant [17].

The following methods are used for managing peri-implantitis individually as well as in combination: decontamination of the implant surface, local debridement, and antimicrobial drugs. Surface decontamination of implants can be done by raising a flap while maintaining and conserving the surrounding soft tissues. The recommended treatment for retrograde peri-implantitis involves surgical debridement of the implant's apical portion, with or without bone substitutes and bone regeneration, and potentially the resection of the implant's apical part [18]. However, it should be noted that instruments softer than titanium, like rubber cups with polishing paste, interdental floss or brushes, and plastic scaling instruments, should be used for the debridement of the implant [19]. The study aimed to evaluate the level of understanding regarding peri-implant among general dentists practicing in Saudi Arabia.

## Materials and methods

Study design and timeframe

From August 2023 to December 2023, a cross-sectional study utilizing a web-based questionnaire was undertaken. The primary focus was on GDPs affiliated with diverse government and private healthcare institutions, as well as dental centers across Saudi Arabia.

Ethical clearance

The Research Ethics Committee of Najran University approved this research under the reference number 202403-076-019314-043901.

Sample size

The sample size was calculated using Cochran's sample size formula, which gives a number of 385. To avoid gender bias, oversampling was done, and the study involved a sample of 460 GDPs in Saudi Arabia, encompassing both male and female dentists. Exclusion criteria comprised consultants, specialists, and undergraduate students.

Methods for quantitative data collection

The collection of quantitative data utilized Google Forms, a template provided by Google, Inc., United States. To ensure singular responses from each participant, the response setting was configured accordingly. Before completing the questionnaire, all study participants were briefed on the study protocol, and written informed consent was obtained. The author used a pre-validated questionnaire created by Barrak and Thomas (2023) [20] that probed participants' awareness of dental implants and associated complications such as peri-implantitis.

The questionnaire comprised two sections. The first section covered demographic data, encompassing gender, practice location, working experience, and level of training. The second section aimed to evaluate participants' knowledge and awareness regarding dental implants and associated complications.

Analysis of data

For coding, tabulation, and analysis of data, IBM SPSS Statistics for Windows, Version 20 (Released 2011; IBM Corp., Armonk, New York, United States) was employed. A chi-square test was utilized to ascertain any statistically significant differences in knowledge based on gender, experience, level of training, and restoring practice. Throughout the analyses, a p-value less than or equal to 0.05 was considered indicative of statistical significance.

## Results

In this cross-sectional study, 460 GDPs actively participated. Notably, 135 individuals (29.3%) identified as female dentists, while the majority, comprising 325 practitioners (70.7%), were male. The distribution across practice settings revealed that 26 participants (5.7%) were affiliated with government hospitals, 181 (39.3%) worked in government dental centers, 19 (4.1%) were associated with private hospitals, 217 (47.2%) operated in private dental centers, 4 (0.9%) were positioned in university hospitals, and 13 (2.8%) practiced in university dental/college centers.

When stratifying the participants based on their professional experience, 156 individuals (33.9%) reported less than three years of experience, 171 (37.2%) had 3-5 years of experience, and 133 (28.9%) had accumulated more than five years of experience. Significantly, 86.5% of the participants hadn’t undergone training in dental implants, distinguishing them from their peers who did receive such training. Within the subset of individuals who received training on implants, 39 practitioners (62.9%) completed short courses, 2 (3.2%) possessed certificates, 2 (3.2%) attained diplomas, 1 (1.6%) participant achieved a Master of Science (MSc) level of training, and 19 (30.6%) dentists underwent alternative forms of training in dental implants. Notably, 454 GDPs (98.7%) were not involved in the restoration or placement of implants, as detailed in Table 1.

**Table 1:** Demographic details of participants

	n	Percentage
Gender		
Female	135	29.3
Male	325	70.7
Total	460	100.0
Current practicing at		
Government hospital	26	5.7
Government dental center	181	39.3
Private hospital	19	4.1
Private dental center	217	47.2
University hospital	4	0.9
University dental/college center	13	2.8
Total	460	100.0
Working experience		
Less than 3 years	156	33.9
3 to 5 years	171	37.2
More than 5 years	133	28.9
Total	460	100.0
Training		
No	398	86.5
Yes	62	13.5
Total	460	100.0
Training Level		
Short course	39	62.9
Certificate	2	3.2
Diploma	2	1.6
MSc	1	1.6
Other	19	30.6
Total	62	13.5
Involved in restoring or placing implants?		
No	454	98.7
Yes	6	1.3
Total	460	100.0

**Figure 1:**
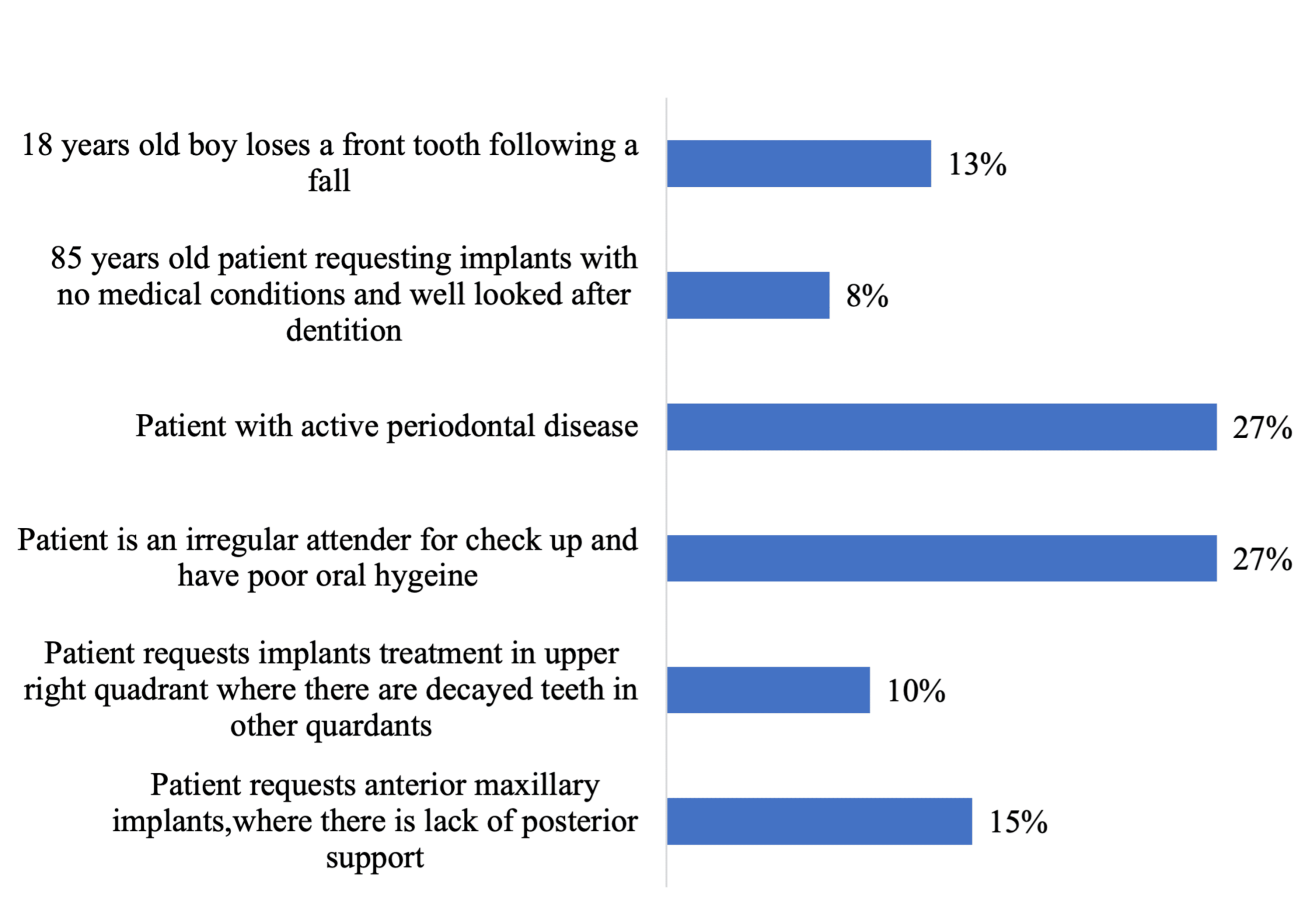
Scenario be appropriate referrals for implant treatment

When participants were asked about the scenario that would be appropriate referrals for implant treatment, two conditions (patients with active periodontal disease and irregular attenders and with poor oral hygiene) were chosen by most of the participants (27% each), followed by 15% of participants who chose patient requests for anterior maxillary implants, where there is a lack of posterior support (Figure 1).

Table 2 provides a comprehensive overview of participant responses regarding dental implants, categorized by gender. A significant proportion of both male (78.8%) and female (74.1%) dentists agreed with the practice of examining dental implants during assessments, even if they were not directly involved in the treatment. Notably, the majority of participants, encompassing 96.5% (28.3% of female dentists and 68.3% of male dentists), demonstrated unawareness of the formal method for diagnosing peri-implant mucositis and peri-implantitis. Further analysis revealed that a considerable number of practitioners (81.3%) engaged in probing activities around implants. Specifically, 103 females (22.4%) and 271 males (58.9%) among the GDPs demonstrated an active involvement in probing procedures related to dental implants. Statistical differences were not present among participants in terms of gender.

**Table 2:** Correlation between gender and knowledge

Response		Gender		Total	Chi-square	p-value
		Female	Male			
Do you specifically examine dental implants during an examination even if you have not carried out the treatment?	No	35 (25.9%)	69 (21.2%)	104 (22.6%)	1.202	0.274
	Yes	100 (74.1%)	256 (78.8%)	356 (77.4%)		
Are you familiar with any formal criteria for diagnosis of peri-implantitis and peri-implant mucositis?	No	130 (28.3%)	314 (68.3%)	444 (96.5%)	0.029	0.865
	Yes	5 (1.1%)	11 (2.4%)	16 (3.5%)		
Do you probe around implants?	No	32 (7.0%)	54 (11.7%)	86 (18.7%)	3.153	0.076
	Yes	103 (22.4%)	271 (58.9%)	374 (81.3%)		

**Figure 2:**
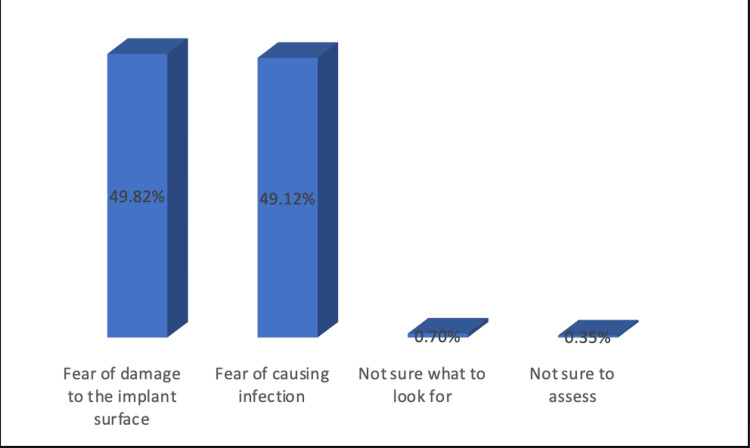
Why you don't probe around the implants?

Among 86 participants who did not probe around implants, 49.8% gave the reason due to fear of implant surface damage, while 49.12% didn’t probe due to fear of infection (Figure 2).

**Figure 3:**
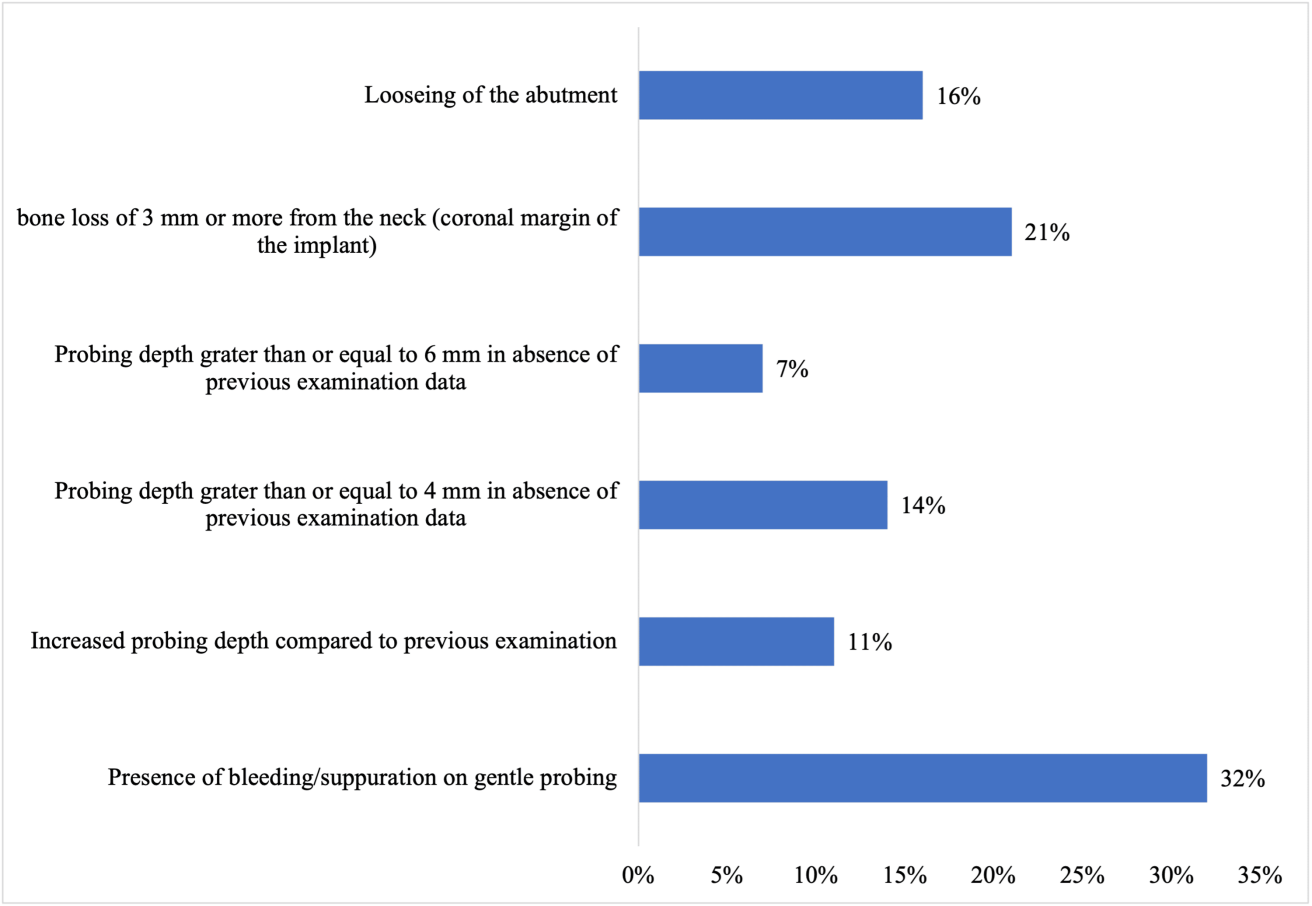
Diagnostic criteria for peri-implantitis

When participants were asked about the criteria for peri-implantitis diagnosis, 32% of dentists chose” bleeding/suppuration on gentle probing,” 21% agreed on “bone loss of 3 mm or more from the neck,” and 16% said that “loosening of the abutment” is the diagnostic criteria (Figure 3).

**Figure 4:**
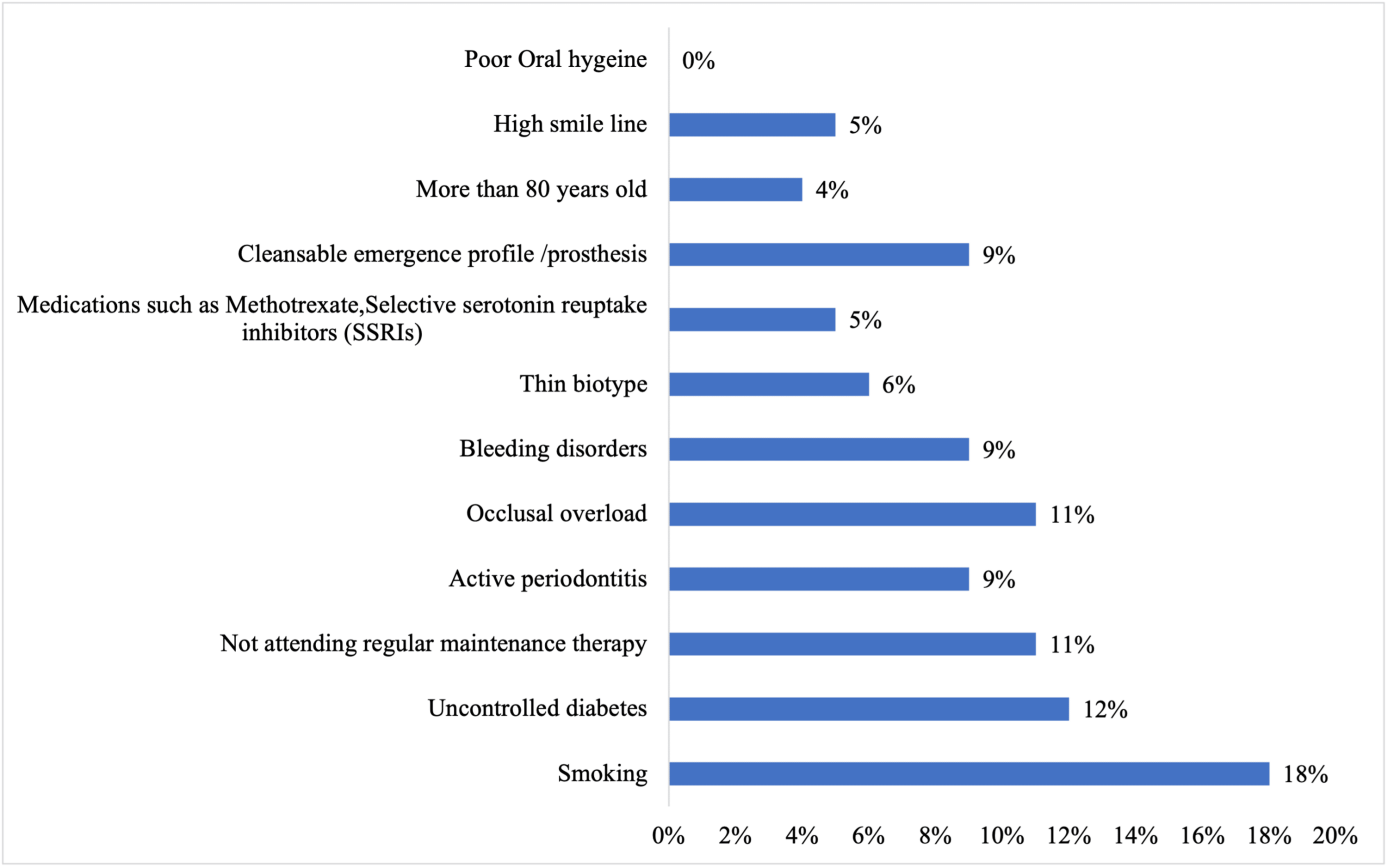
Major factors to predispose to peri-implantitis

The factors that prompt peri-implantitis are presented in Figure 4. Eighteen percent of GDPs chose smoking, followed by uncontrolled diabetes (12%), occlusal overload (11%), non-regular maintenance therapy (11%), active periodontitis (9%), bleeding disorders (9%), cleansable emergence profile (9%), and thin biotype (6%).

The correlation between working experience and awareness of participants is described in Table 3. Most of the participants from each group (114 (24.8%), 136 (29.6%), and 106 (23.0%) with less than three years, 3-5 years, and more than five years of experience, respectively) specifically examined dental implants during the examination. Only 16% of participants from all three groups (1.5%, 0.7%, and 1.3%) were familiar with the formal criteria for diagnosis of peri-implantitis. One hundred twenty-six (27.4%) participants with experience of less than three years, 134 (29.1%) with 3-5 years of experience, and 114 (24.8%) with more than five years of experience were involved in probing around implants. This relationship didn’t show any significant effect.

**Table 3:** Correlation between working experience and knowledge

Response		Experience			Total	Chi-square	p-value
		Less than 3 years	3-5 years	More than 5 years			
Do you specifically examine dental implants during an examination even if you have not carried out the treatment?	No	42 (9.1%)	35 (7.6%)	27 (5.9%)	104 (22.6%)	2.512	0.285
	Yes	114 (24.8%)	136 (29.6%)	106 (23.0%)	356 (77.4%)		
Are you familiar with any formal criteria for diagnosis of peri-implantitis and peri-implant mucositis?	No	149 (32.4%)	168 (36.5%)	127 (27.6%)	444 (96.5%)	2.409	0.300
	Yes	7 (1.5%)	3 (0.7%)	6 (1.3%)	16 (3.5%)		
Do you probe around implants?	No	30 (6.5%)	37 (8.0%)	19 (4.1%)	86 (18.7%)	2.705	0.259
	Yes	126 (27.4%)	134 (29.1%)	114 (24.8%)	374 (81.3%)		

Forty-seven participants (10.2%) who received training about dental implants agreed that they examine the dental implants even if they have not done this treatment. While 309 (67.3%) dentists who didn’t receive any kind of training responded the same. Only two (0.4%) participants who had training and 14 (3.1%) who didn’t have training were aware of the formal method for diagnosis of peri-implant mucositis and peri-implantitis. Most of the participants from both groups (training and no training) were involved in probing around dental implants. There was no significant difference among those who received training and those who didn’t (Table 4).

**Table 4:** Correlation between training and knowledge

Response		Training		Total	Chi-square	p-value
		No	Yes			
Do you specifically examine dental implants during an examination even if you have not carried out the treatment?	No	89 (19.4%)	14 (3.1%)	103 (22.4%)	0.011	0.918
	Yes	309 (67.3%)	47 (10.2%)	356 (77.6%)		
Are you familiar with any formal criteria for diagnosis of peri-implantitis and peri-implant mucositis?	No	384 (83.3%)	59 (12.9%)	443 (96.5%)	0.009	0.925
	Yes	14 (3.1%)	2 (0.4%)	16 (3.5%)		
Do you probe around implants?	No	81 (17.6%)	5 (1.1%)	86 (18.7%)	5.133	0.023
	Yes	317 (69.1%)	56 (12.2%)	373 (81.3%)		

Table 5 outlines the findings from 62 participants who underwent training on dental implants. Of those surveyed, 28 (6.1%) individuals with a short course background, 1 (0.2%) with certificates, 1 (0.2%) from diploma programs, 1 (0.2%) with MSc qualifications, and 17 (3.7%) with other forms of training expressed that they routinely examine dental implants during evaluations, even in the absence of treatment. Across all levels of training, over 96.8% of participants demonstrated no familiarity with formal criteria for diagnosing peri-implantitis. Notably, only five (8.1%) respondents admitted to not probing around implants, a statistically non-significant finding (p=0.049).

**Table 5:** Correlation between level of training and knowledge

Response		Specialty					Total	Chi-square	p-value
		Short course	Certificate	Diploma	MSc	Other			
Do you specifically examine dental implants during an examination even if you have not carried out the treatment?	No	11 (2.4%)	1 (0.2%)	0	0	2 (0.4%)	14 (22.6%)	3.725	0.590
	Yes	28 (6.1%)	1 (0.2%)	1 (0.2%)	1 (0.2%)	17 (3.7%)	48 (77.4%)		
Are you familiar with any formal criteria for diagnosis of peri-implantitis and peri-implant mucositis?	No	37 (8.0%)	2 (0.4%)	1 (0.2%)	1 (0.2%)	19 (4.1%)	60 (96.8%)	1.147	0.950
	Yes	2 (0.2%)	0	0	0	0	2 (3.2%)		
Do you probe around implants?	No	5 (33.9%)	0	0	0	0	5 (8.1%)	6.893	0.229
	Yes	34 (7.4%)	2 (0.4%)	1 (0.2%)	1 (0.2%)	19 (4.1%)	57 (91.9%)		

The correlation between restorative practice and awareness of peri-implantitis is detailed in Table 6. Among those not engaged in restoration, 76.3% acknowledged examining dental implants during evaluations, while 1.1% of those involved in restoration shared the same perspective. An overwhelming majority in both groups, only no restoring group (3.3%) were acquainted with formal diagnostic criteria, and this discrepancy was statistically non-significant (p=0.237). Additionally, nearly 81.3% of participants from both groups reported actively participating in probing around implants.

**Table 6:** Correlation between restoring and knowledge

Response		Restoring		Total	Chi-square	p-value
		No	Yes			
Do you specifically examine dental implants during an examination even if you have not carried out the treatment?	No	103 (22.4%)	1 (0.2%)	104 (22.6%)	0.123	0.726
	Yes	351 (76.3%)	5 (1.1%)	356 (77.4%)		
Are you familiar with any formal criteria for diagnosis of peri-implantitis and peri-implant mucositis?	No	439 (95.4%)	5 (1.1%)	444 (96.5%)	3.150	0.076
	Yes	15 (3.3%)	1 (0.2%)	16 (3.5%)		
Do you probe around implants?	No	86 (18.7%)	0	86 (18.7%)	1.398	0.237
	Yes	368 (80.0%)	6 (1.3%)	374 (81.3%)		

## Discussion

This research sheds light on the knowledge of GDPs in Saudi Arabia regarding peri-implant diseases. The participants surveyed exhibit sufficient understanding in certain areas but demonstrate less than optimal knowledge in others.

An important focus in contemporary dentistry revolves around the biological complications linked to dental implant therapy. Typically of an inflammatory nature, these complications often involve bacterial challenges, as highlighted by various studies [21-23]. Peri-implantitis is a frequently recognized clinical condition, distinguished by an inflammatory lesion in the mucosa surrounding the implant [12]. Although implants typically exhibit high success rates, there is a growing occurrence of peri-implantitis documented in scholarly literature [6]. This emphasizes the importance for general practitioners to improve their understanding of the prevention, diagnosis, and management of these conditions. Consequently, ongoing education becomes imperative for the continued advancement of their professional expertise.

The British Society of Periodontology and Implant Dentistry (BSP) has provided strategies outlining the referral pathway for patients with peri-implantitis [24]. Various parameters, such as suppuration, bone loss, bleeding, and probing depth, are employed to determine the onset, extent, and severity of peri-implantitis, aiding in its diagnosis. Mombelli et al. [25] described bone loss in the surrounding implants as a distinct, crater-like fault without significant indications of implant mobility. Research conducted by Barrak and Thomas revealed that 68.3% of participants were not acquainted with the criteria of diagnosis for peri-implantitis. This deficiency in awareness might result in inadequate supervision or delayed referral and treatment. A noteworthy finding was that a considerable proportion of participants linked bleeding upon probing with peri-implantitis, acknowledging it as one of the diagnostic indicators for peri-implant diseases. The study highlighted a considerable number of correct answers regarding other diagnostic criteria, particularly acknowledging the significant depth of probing (6 mm or more) and bone level [20]. In our study, 32% of dentists selected "presence of bleeding/suppuration on gentle probing" as a diagnostic criterion, while 21% agreed on "bone loss of 3 mm or more from the neck." Additionally, 16.5% indicated that "loosening of the abutment" was a diagnostic criterion for peri-implantitis.

Möst et al. [26] investigated the efficacy of a training program related to dental implants designed to augment the understanding of dental students. Their results suggested that the group with three years of training achieved higher scores in fundamental implant design and information in comparison to the group with three days of training. A survey conducted in 2008 among dental schools in Ireland and the UK showed that while a significant proportion of schools (87%) included education related to implants in their undergraduate programs, only 46% offered practical training in planning for implant treatment and observation of restoration [27].

The survey delved into the awareness regarding peri-implantitis risk factors among GDPs. In research by Barrak and Thomas, a substantial percentage (94.2%) identified periodontitis as a risk factor for peri-implantitis. Likewise, bad oral hygiene was recognized as a risk factor by a significant majority (94.6%) of participants. This indicates an understanding among participants regarding the impact of plaque in starting peri-implantitis [28], given its significance as an etiological factor. Uncontrolled diabetes and smoking were also acknowledged as factors for disease by a majority of respondents (93.6% and 94.2%, respectively) [24]. The link between smoking and periodontitis, tooth, and attachment loss is widely recognized, with research indicating a possible extension of this impact to tissues of peri-implant. Karoussis et al. found that over a period of 10 years, 18% of implants in smokers developed peri-implantitis, while only 6% did so in nonsmokers [29]. However, contradictory results from cross-sectional studies indicate that smokers may not face a higher risk of peri-implantitis [30-32].

Schwarz et al. resolved that there is presently no definitive sign establishing smoking as a decisive risk factor for peri-implantitis [28]. In the present study, 18% of GDPs identified smoking as a contributing factor, followed by uncontrolled diabetes (12%), occlusal overload (11%), non-regular maintenance therapy (11%), active periodontitis (9%), bleeding disorders (9%), cleansable emergence profile (9%), and thin biotype (6%) as factors associated with peri-implantitis.

## Conclusions

The findings of this investigation suggest that the majority of participants do not possess sufficient knowledge regarding the mechanisms, initiation, and progression of periodontitis and periimplantitis, as well as their management. Notably, a considerable number of practitioners (18.7%) do not conduct probing around implants, Nonetheless, there is no statistically significant difference observed among participants based on gender, experience, and training level. The study emphasizes the necessity for additional continuing professional development courses provided by educational institutions, focusing on improving criteria for diagnosis, appropriate situations for implant recommendation, and understanding risk factors linked with peri-implantitis to enhance the overall care of patients. Furthermore, it highlights the need for incorporating peri-implant disease diagnosis into undergraduate training programs.

## Appendices

**Table 7:** Questionnaire

S. No.	Question
DEMOGRAPHIC DATA	
1.	Gender - Male Female
2.	You current practicing at - Government Hospital Government Dental Center Private Hospital Private Dental Center University Hospital University Dental/College Center
3.	Working experience - Less than 3 years 3 to 5 years More than 5 years
KNOWLEDGE	
4.	Have you ever received any training about implants? Yes No
5.	What is the level of training? Short course Certificate Diploma MSc Other:
PRACTICE	
6.	Are you currently involved in restoring or placing implants? Yes No
7.	Would the following scenario be appropriate referrals for implant treatment (can choose multiple answers) Patient requests anterior maxillary implants, where there is lack of posterior support. Patient requests implants treatment in upper right quadrant where there are decayed teeth in other quadrants. Patient is an irregular attender for check up and have poor oral hygiene. Patient with active periodontal disease. 85 years old patient requesting implants with no medical conditions and well looked after dentition. 18 years old boy loses a front tooth following a fall.
8.	Do you specifically examine dental implants during an examination even if you have not carried out the treatment? Yes No
9.	Are you familiar with any formal criteria for diagnosis of peri-implantitis and peri-implant mucositis? Yes No
10.	Do you probe around implants? Yes No
11.	Why you don't probe around the implants? Fear of damage to the implant surface. Fear of causing infection. Fear of becoming involved medico-legally. Not sure what to look for. Not sure to assess.
12.	Which of the following are diagnostic criteria for peri-implantitis? Presence of bleeding/suppuration on gentle probing. Increased probing depth compared to previous examination. Probing depth greater than or equal to 4 mm in absence of previous examination. Probing depth greater than or equal to 6 mm in absence of previous examination. Loosening of the abutment. Bone loss of 3 mm or more from the neck (coronal margin of the implant).
13.	From the list below, choose the major factors to predispose to peri-implantitis Smoking. Uncontrolled diabetes. Not attending regular maintenance therapy. Active periodontitis. Occlusal overload. Bleeding disorders. Thin biotype. Medications such as Methotrexate, Selective serotonin reuptake inhibitors (SSRIs). Cleansable emergence profile /prosthesis. More than 80 years old. High smile line. Poor oral Hygiene.
